# Specific Effects of Anti-Hypertensive Treatment in an Older Patient with Dementia

**DOI:** 10.2174/1874205X01711010015

**Published:** 2017-04-17

**Authors:** Jadwiga Attier Zmudka, Jean Marie Sérot, Salif Dao, Claire Sorel, Anne-Sophie Macaret, Olivier Balédent

**Affiliations:** 1 Department of Gerontology, General Hospital Saint Quentin, BioFlowImage, University of Picardy Jules Verne, Amiens, France; 2 Department of Radiology, General Hospital Saint Quentin, Saint Quentin, France; 3 Department of Cardiology, General Hospital Saint Quentin, Saint Quentin, France; 4 Department of Neurology, General Hospital Saint Quentin, Saint Quentin, France; 5 BioFlowImage Image processing unit, University Hospital & University of Picardy Jules Verne, Amiens, France

**Keywords:** Hypertension, Dementia, Cerebral blood flow, Stroke, Asprin, MRI

## Abstract

Dementia is one of the most common health problems in the world. Alzheimer’s disease (AD) is the most common form of dementia. The presence of vascular risk factors such as hypertension (HT) may increase the risk of AD [1,2]. The relation between blood pressure (BP) and dementia has been the subject of numerous epidemiological studies, midlife HT is a risk factor for dementia and AD [3-7] but the association between HT and risk of dementia is lower in the older population [8].

A fair modulation of an antihypertensive treatment, based on the cognitive status of the elderly, can avoid multiple complications.

A case of an older for whom cognitive improvement and reduced risk of falls were noticed after mild blood pressure elevation is reported.

## CASE REPORT

An 88-year-old woman living alone at home was consulted for cognitive decline. She presented hypertension for more than 40 years, diagnosed after her menopause. She had also coronary disease, osteoporosis, osteoarthritis and dyslipidemia. Her treatment was olmesartan 20 mg every two days (which is not standard according to guidelines), since 7 years (her previous treatment with angiotensin-converting enzyme inhibitors was replaced due to cough), bisoprolol 10 mg, nicorandil 10mg, aspirin 75 mg, ezetimibe 10 mg, cholecalciferol and alendronic acid 70 mg/2800 UI, fentanyl 12 µg every 3 days. Drugs were administrated by a visiting nurse. There was no modification in her treatment for more than one year. The medical questioning showed that the patient did not know the reason for our consultation, her birth date nor the number of her children. Her daughter revealed that the patient was an insomniac. Her neighbors found her few times lost outside her home.

The clinical examination showed an extrapyramidal rigidity without tremor.

She had no visual troubles, but had hearing loss. The geriatric scales showed a Charlson score [9] at 4, activities of daily living (ADL) [10] at 4/6, instrumental activities of daily living (IADL) [11] at 1/8, timed up and go test was not performed as the walk and equilibrium were altered. The primary cognitive assessment revealed a severe cognitive decline with Mini-Mental State Examination (MMSE) [12] at 11/30, the confusion assessment method (CAM) [13] was at 0/4 and mini geriatric depression scale (miniGDS) [14] at 2/4. The clinical dementia rating (CDR) [15] was at 3.

During hospitalization in acute geriatrics unit, we noted repeated falls with traumatic consequences. Falls occurred mostly in the morning, related to low blood pressure (by 110/60).

The orthostatic hypotension test was negative many times, excluding an autonomic failure.

Neuropsychological assessment (MMSE, clock test, trail making test, Mattis dementia rating scale, Rey-Osterrieth complex figure) confirmed dementia, according to DSM IV criteria [16]. There was no hallucination or variability of cognitive tests. Cognitive profile and neuroimaging abnormalities suggested a mixed dementia [17], vascular part involving executive alterations and impaired processing speed and hippocampal part related to the episodic memory deficit.

24 h rhythmic monitoring showed normal sinus rhythm with minor disorders like ventricular extrasystole, not sufficient to justify the falls.

24 h blood pressure monitoring showed a mean arterial pressure by 120/53 mmHg, based only on the day period; monitoring was not available by night due to behavioral disturbances. The minimal values achieved as 102/43 mm Hg.

**Fig. (1):**
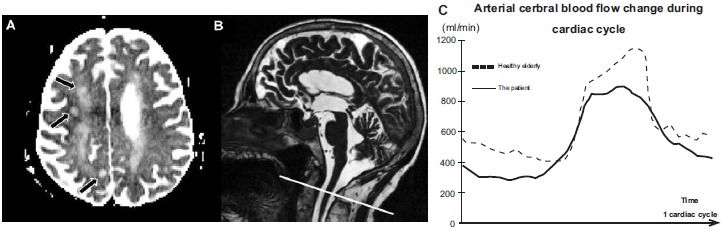
**A:** The T2* Magnetic Resonance Imaging shows three areas of semi recent ischemia in the white matter, including: frontal right posterior, right posterior transitional zone and beside the left central sulcus. **B:** A sagittal T2 sequence was used as localizers to select the anatomical levels for the Phase Contrast Magnetic Resonance Imaging used for the flow quantification over the cardiac cycle (CC). Acquisition plane was selected to be perpendicular to the vertebral and internal carotid arteries. **C:** The graph shows the curves of the mean cerebral arterial blood flows, for the patient and a healthy elderly subject. This quantitative acquisition shows a decrease of pulsatility of the arterial cerebral blood flow of the patient over the cardiac cycle.

Magnetic resonance imaging (MRI) showed three areas of semi recent ischemia (roughly 3 weeks ago). Vascular alterations were presented by periventricular and deep white matter leukoencephalopathy (Fazekas II), and it was hippocampal atrophy (Scheltens IV). A phase contrast MRI flow sequence (PCMRI), measuring the cerebral arterial circulation [18], performed in a research purpose, showed a decrease in arterial pulsatility (Fig. **1**). This finding could be related to a hypotension as demonstrated by the blood pressure monitoring.

We think that the dementia syndrome occurred earlier as her daughter reported minor mnesic problems for few years. We hypothesized that the multiple strokes were caused by low cerebral blood flow in patient treated with antihypertensive drugs in her midlife, her antihypertensive treatment being the same for many years. The diastolic hypotension supported this hypothesis. However, this patient had long-term high blood pressure, dyslipidemia and a history of coronary heart disease and the hypothesis of thromboembolic mechanisms could not be formally eliminated.

In an aim to preserve the quality of life of our patient, antihypertensive treatment with the sartans was stopped and β-blockers decreased to 2.5 mg. The mean arterial blood pressure increased up to 150/90 mm Hg. Moreover, the MMSE improved to 16/30 and the patient did not fall anymore during more than one year after.

## DISCUSSION

Hanon's study indicated a significant decrease in BP in patients with AD after one year of follow up, independently of age, gender, body mass index and antihypertensive therapy. The largest decrease in BP was observed in patients with the severest impairment in dementia at the baseline, suggesting that blood pressure decrease seems to be mainly a secondary phenomenon in Alzheimer's disorders [19]. The decrease in BP could increase amyloid genesis and taupathy strategic locations of the brain that regulate BP. Recent study [20] showed also reduced brain circulation leading to continuous hypoperfusion that increases generation β-amyloid peptides particularly APO-ε4 carriers and thus mildly elevated blood pressure seems to benefit mental and physical functions in these older patients. The APO E haplotype of our patient was unavailable; this is a limitation of our study.

The mean arterial blood pressure by 150/90 obtained after drugs modification was closer to guideline recommendations in elderly [21, 22]. However, the elderly in upper age are rarely included in major hypertension studies.

The PC-MRI flow sequence is rapid (around 2 minutes), doesn’t require an injection and brings quantitative information of the cerebral arterial flow over the cardiac cycle. Nevertheless, this blood flow investigation is still not used commonly in neurodegenerative pathology.

In conclusion, we report the case of a patient with a severe cognitive decline, secondary to multiple strokes caused by low cerebral blood flow in patient treated with antihypertensive drugs since her midlife, which have not been modified when the first signs of memory loss appeared. The decrease of antihypertensive drugs improved her cognitive functions and protected her from falling consequences.

Physicians who deal with patients suffering from neurodegenerative diseases must keep in mind that the antihypertensive medication should be reevaluated.

## AUTHOR CONTRIBUTIONS

All authors had full access to all of the data in the study and take responsibility for the integrity of the data and the accuracy of the data analysis. Attier-Zmudka: Medical care, case reporter, drafting of manuscript, critical revision of manuscript. .

Sérot: Supervision, critical revision of manuscript.

Dao: Interpretation of magnetic resonance imaging.

Sorel: Interpretation of rhythmic monitoring

 Macaret: Neuropsychological statement interpretation. 

Balédent: Administrative, technical, material support, supervision and interpretation of imaging flow data. 

##  SPONSOR’S ROLE

None.

## Figures and Tables

**Table:** 

**Elements of Financial/Personal Conflicts**	**JAZ**		**JMS**		**SD**		**CS**		**ASM**		**OB**	
	**Yes**	**No**	**Yes**	**No**	**Yes**	**No**	**Yes**	**No**	**Yes**	**No**	**Yes**	**No**
**Employment or Affiliation**												
		**x**		**x**		**x**		**x**		**x**		**x**
**Grants/Funds**												
		**x**		**x**		**x**		**x**		**x**		**x**
**Honoraria**												
		**x**		**x**		**x**		**x**		**x**		**x**
**Speaker Forum**												
		**x**		**x**		**x**		**x**		**x**		**x**
**Consultant**												
		**x**		**x**		**x**		**x**		**x**		**x**
**Stocks**												
		**x**		**x**		**x**		**x**		**x**		**x**
**Royalties**												
		**x**		**x**		**x**		**x**		**x**		**x**
**Expert Testimony**												
		**x**		**x**		**x**		**x**		**x**		**x**
**Board Member**												
		**x**		**x**		**x**		**x**		**x**		**x**
**Patents**												
		**x**		**x**		**x**		**x**		**x**		**x**
**Personal Relationship**												
		**x**		**x**		**x**		**x**		**x**		**x**
